# Mapping healthcare service utilisation for diabetes and hypertension in Tanzania mainland: bayesian spatial modeling

**DOI:** 10.3389/frhs.2026.1791146

**Published:** 2026-05-22

**Authors:** Elia Magwaja, Jim Todd, Ladislaus Chang’a, Anthony Kapesa, Eustasius Musenge, George PrayGod, Brenda Kitilya, Eliud Kamala, Eliakimu Kapyolo, Daniel Faurholt-Jepsen, Zvifadzo Matsena Zingoni

**Affiliations:** 1 College of Social Sciences, University of Dar es Salaam, Dar es Salaam, Tanzania; 2 Mwanza Research Centre, National Institute for Medical Research, Mwanza, Tanzania; 3 School of Public Health, Catholic University of Health and Allied Sciences, Mwanza, Tanzania; 4 Tanzania Meteorological Authority, Dodoma, Tanzania; 5 Division of Epidemiology and Biostatistics, School of Public Health, Faculty of Health Sciences, University of the Witwatersrand, Johannesburg, South Africa; 6 Muhimbili Medical Research Centre, National Institute for Medical Research, Dar es Salaam, Tanzania; 7 Department of Infectious Disease, Rigshospitalet, Copenhagen, Denmark; 8 Department of Clinical Medicine, University of Copenhagen, Copenhagen, Denmark; 9 Center for Biomedical Modeling, Semel Institute for Neuroscience & Human Behavior, Department of Psychiatry, UCLA David Geffen School of Medicine, Los Angeles, CA, United States

**Keywords:** Bayesian model, diabetes, hypertension, non-communicable diseases, spatial mapping, Tanzania

## Abstract

Diabetes and hypertension pose a growing non-communicable disease (NCD) burden in sub-Saharan Africa. However, in most countries, healthcare service provision remains uneven and there are no comprehensive methods for monitoring service use. We mapped and modeled district-level patient healthcare service utilisation for diabetes and hypertension in mainland Tanzania to identify spatial patterns and socio-demographic correlates using Bayesian spatial models. We analyzed district-level diabetes and hypertension healthcare service utilisation rates collected from health care facilities in 2024. All 184 districts of Tanzania mainland were included. Huge geographic disparities in healthcare service utilisation were observed across different districts. Diabetes and hypertension healthcare service utilisation were consistently highest in urban districts such as Kinondoni, Ilala, Arusha urban, and Iringa urban, and lowest in rural districts such as Namtumbo, Tunduru, Sikonge, and Mlele. Significant spatial clustering was detected for hypertension (Moran's I = 0.173, *p*-value = 0.003), and weaker but statistically significant clustering for diabetes (Moran's I = 0.099, *p*-value = 0.025). For hypertension, stronger clustering was observed among females (Moran's I = 0.195, *p*-value < 0.001). Being literate had a 3% increased rate of healthcare service utilisation for both diabetes [incidence rate ratio (IRR) = 1.03; 95% credible interval (CrI): 1.01–1.05] and hypertension (IRR = 1.03; 95%CrI: 1.01–1.04). Owning a mobile phone showed an increased rate in healthcare service utilisation of 10% (IRR = 1.10, 95%CrI: 1.06–1.14) for diabetes and 6% (IRR = 1.06; 95%CrI: 1.02–1.10) for hypertension. Ownership of television had a 2% increased rate (IRR = 1.02; 95%CrI 1.01–1.05) for diabetes healthcare service utilisation, while owning a radio showed a 3% (IRR = 0.97, 95%CrI: 0.96–0.99) decreased rate in diabetes healthcare service utilisation. Household size showed a 22% (IRR = 0.78; 95%CrI: 0.64–0.94) reduction in hypertension healthcare service utilisation rate. Overall, Tanzania mainland exhibits marked spatial and gender-specific inequalities in diabetes and hypertension healthcare service utilisation. These inequalities are driven by urban development, with urban districts consistently showing higher healthcare service utilisation, while rural districts, particularly those in the southern and western parts of the country, remain underserved. These findings underscore the need for spatially targeted NCD interventions and district-level resource allocation to ensure equitable access to chronic disease care.

## 1 Introduction

Diabetes and hypertension are two important non-communicable diseases (NCDs) contributing to 41 million deaths every year (1), with diabetes contributing 6.7 million deaths in adults aged 20–79 years in 2021 (2). Statistics from the International Diabetes Federation (IDF) show that, until 2021, more than 540 million adults (aged 20–79 years) were living with diabetes, and more than 240 million had undiagnosed diabetes (2). The projected number of people living with diabetes will be over 640 million in 2030 and 780 million in 2045 (2). Hypertension, on the otherhand, is the leading cause of morbidity and premature mortality in the world (3). In 2023, the World Health Organization (WHO) estimated that about 1.3 billion people aged 30–79 years were living with hypertension worldwide (3), with two-thirds of these people living in low- and middle-income countries (LMICs). Yet, 46% were unaware of the condition, and only 42% of the adults diagnosed were on treatment (3). This combined effect of the two conditions threatens the implementation of the 2030 Sustainable Development Agenda, which includes reducing premature mortality from NCDs by one-third by 2030 (Sustainable Development Goal 3, Target 3.4) (4).

The rising burden of NCDs in LMICs is largely driven by urbanisation, changing lifestyles, and weak and under-resourced health systems (5–10). In Sub-Saharan Africa, there is a steadily rising demand for diagnostic services and chronic care for both diabetes and hypertension (11). According to the IDF, about 75% of the people living with diabetes are from the LMICs. In Africa alone, more than 24 million adults had diabetes, and more than 400,000 died of diabetes complications in 2021 (2). In Tanzania, the annual diabetes-related deaths among adults (aged 20–79 years) rose from approximately 6,000 people in 2011 to about 36,000 people in 2021 (2), while hypertension contributed about 26% to 40% of annual deaths (12, 13). A Tanzanian national survey conducted in 2013 among those aged 25–64 years reported hypertension as the most prevalent NCD in Tanzania, with a prevalence of 25.9% and diabetes at 9.1% (14). High prevalence rates of diabetes and hypertension have also been reported in studies conducted in different parts of Tanzania among adults aged at least 18 years (15–18). More recent estimates suggest an even higher prevalence of diabetes and hypertension, especially in urban settings, where access to processed foods and a sedentary lifestyle is more common (19).

The rising burden of diabetes and hypertension has been linked to several risk factors, including obesity, physical inactivity, poor diet, genetic susceptibility, and socioeconomic barriers, including low education and limited access to healthcare (11, 20–23). Furthermore, there is growing evidence that environmental factors such as extreme weather and climate, and air pollution are associated with NCDs (24–26). The necessity of incorporating spatial elements in NCD planning is highlighted by the spatial and contextual disparities in NCD prevalence caused by economic, geographic, and health care service inequalities (27).

Diabetes and hypertension can be effectively managed using well-established and affordable medications. Nevertheless, in many low-resource countries, substantial gaps persist in long-term access to care, early diagnosis, and preventive measures. As a result, most at-risk populations remain unaware of their medical conditions (27). For example, a study conducted in rural Morogoro, Tanzania, found that about 29% of individuals aged 24–64 years were hypertensive; however, only about 34% were aware of their condition, and only about 35% of those who were aware were receiving medication (28). Similar patterns are observed for diabetes, where patients often seek treatment and care only after complications have developed. These challenges are exacerbated by a healthcare system that continues to put more efforts towards infectious disease prevention and control, while access to chronic NCD care remains uneven. As a result, many NCD patients remain undiagnosed or lack routine follow-up, particularly in underserved or rural settings, making disease treatment a major challenge. If appropriate preventive measures are not well designed, countries in East Africa (including Tanzania) will continue to experience an increase in these NCD incidences (29).

Few studies have examined healthcare service utilisation patterns using spatial approaches (56). Majority of current research has identified individual or household-level factors to be associated with diabetes and hypertension and ignored spatial patterns. To understand how geographic location, sociodemographic characteristics, and healthcare accessibility influence healthcare-seeking behavior, spatial epidemiological studies are important (57). For instance, studies examining geographic patterns of chronic disease care and hypertension management in Sub-Saharan Africa and other LMICs, have revealed substantial regional inequalities in diagnosis, treatment, and healthcare access (27, 30). These studies highlight the importance of contextualized spatial analysis for guiding health service planning, especially in LMICs. Therefore, understanding the spatial patterns of NCD service utilisation is important in resource allocation at local levels and offers important insights into healthcare seeking behavior patterns. Moreover, the majority of research in Tanzania is based on cross-sectional national surveys and therefore lacks district-level information. However, regular health data gathered through the District Health Information System 2 (DHIS2) offers an opportunity for real-time, geographical analysis of healthcare service utilisation (31). Given the difference in healthcare infrastructure, socio-economic status, and environmental exposures across Tanzania mainland, the use of spatial analysis using Bayesian Hierarchical Models (BHMs) is ideal. These models are appropriate for mapping the geographic distribution of diabetes and hypertension service utilisation while accounting for spatial dependency and uncertainty. The models are also suitable for identifying spatial patterns of care and supporting equitable health planning.

The aim of this study was, therefore, to map the spatial distribution of healthcare service utilisation for diabetes and hypertension across all districts in mainland Tanzania for the year 2024 using DHIS2 data. The goal was to identify geographic disparities in healthcare service utilisation and link these to district characteristics to inform health system strengthening, NCD program planning, and future epidemiological research. This is a PhD study nested within the impact of a variable climate on long-term adverse health effects in Tanzania (CLIMATCH) study, which investigates the relationship between climate change and non-communicable diseases in Tanzania.

## 2 Methods

### 2.1 Study design and data source

This study employed a cross-sectional ecological spatial analysis of healthcare service utilisation for diabetes and hypertension across all 184 districts of mainland Tanzania. Healthcare service utilisation was defined as the use of healthcare services, measured by the number of routine patients' visits for diabetes and hypertension monitoring. Data on patient visits for both conditions were obtained from the DHIS2 for the year 2024. DHIS2 is used for routine health data reporting, including patient visits for diabetes and hypertension. The dataset included district-level counts of patient visits for diabetes and hypertension. The district boundary shapefiles were obtained from the National Bureau of Statistics, allowing linkage of service data to spatial units for mapping and spatial analysis.

Population indicators for each district were extracted from the most recent national census (2022) as provided by the National Bureau of Statistics (NBS). The 2022 national census provides the most recent official district-level population data available. Independent district characteristics included district population size measured as number of people in a district, rural and urban classification of the district, percentage of education attainment at district level (literacy rate), access to mass media as measured by ownership of radios, televisions and mobile phones, district household size, and healthcare access as measured by the proportion of health facilities in each district. All these variables were extracted from the 2022 population census.

Prior to data analysis, the DHIS2 dataset was reviewed to assess data completeness and consistency across districts. This included inspection of missing values, verification of district identifiers, and screening for inconsistent patient visit counts for diabetes and hypertension. The completeness of the variables used in the analysis was also assessed across all districts. No districts were found to have missing values for the variables used in the study. Since the data were obtained from routine health facility reporting in DHIS2, utilisation counts represent outpatient visits recorded by health facilities for the two conditions. Although no missing values were detected at the district level, routine health information systems may still be subject to reporting variability across facilities. The dataset was therefore carefully reviewed for potential inconsistencies before proceeding with the data analyses.

### 2.2 Data analysis

All data processing was performed using R version 4.4.1. The spdep and sf R packages were used for this analysis.

#### 2.2.1 Descriptive statistics

Healthcare service utilisation was standardized into rates per 1,000 population, taken from the NBS estimation of the size of the population in each district in 2024. Continuous variables were expressed as mean ± standard deviation (SD) for normally distributed variables, and median and interquartile range (IQR) for non-normally distributed variables.

#### 2.2.2 Choropleth mapping

Descriptive maps for diabetes and hypertension were generated to show observed spatial patterns of healthcare service utilisation rates per 1,000 population at the district level. These maps were generated for the national estimates for both diabetes and hypertension and were generated for gender-disaggregated estimates for hypertension healthcare service utilisation only, as they revealed distinct spatial patterns not evident in the aggregated national maps.

#### 2.2.3 Spatial autocorrelation and hotspot analysis

We tested for global spatial autocorrelation using Moran's I statistic, which measures the degree of spatial clustering. A positive Moran's I suggests that neighboring districts have similar values, indicating the presence of spatial clustering of healthcare service utilisation in Tanzania mainland (32). A statistically significant Moran's I (*p* < 0.05) led to the rejection of the null hypothesis, “healthcare service utilisation is randomly distributed”, and hence a justification for subsequent spatial analyses.

Only variables with statistically significant Moran's I (*p* < 0.05) were retained for further hotspot analysis using the Getis_Ord Gi* statistics. Hotspot analyses were performed to identify local clusters of high or low healthcare service utilisation. Hotspot districts were identified for national and gender-specific hypertension healthcare service utilisation, as well as for national and female diabetes healthcare service utilisation. Queen contiguity weights (33) were used to define neighboring districts, and statistical significance was assessed at a 95% confidence level.

#### 2.2.4 Bayesian hierarchical spatial modeling

To quantify spatial variation and assess potential covariate effects, Bayesian hierarchical models were implemented using the Integrated Nested Laplace Approximation (INLA) framework in R using the INLA package. This was implemented under the Negative Binomial family, where the rates for healthcare service utilisation for diabetes and hypertension were analyzed separately. Modeling proceeded in two stages: firstly, the non-spatial models, which included district-level covariates but ignored spatial dependency, and secondly, the spatial models incorporating both structured (spatially correlated) and unstructured (independent) random effects to account for spatial autocorrelation (34, 35). The spatial models were then performed using the Besag-York-Mollié 2 (BYM2), which considers that observations in neighboring areas may have more similar observations than in areas farther away (58, 59). Four models were run for each NCD: model 1 (no spatial effects model), model 2 (unstructured spatial effects model), model 3 (structured spatial effects model), and model 4 [convolutional (structured and unstructured spatial effects)] model. The fixed effects of the model estimates were reported as Incidence Rate Ratios (IRRs) and 95% credible intervals (CrI).

Model selection was based on diagnostic values, the deviance information criterion (DIC), and the Watanabe-Akaike information criterion (WAIC). The model with the lowest values of DIC/WAIC was considered the best-fitting (36, 37). Predicted values from the combined model were mapped to visualize district-level deviations from the national average healthcare service utilisation rate. Bayesian spatial modeling focused on aggregated national estimates to assess residual spatial structure after adjusting for covariates. Since the goal was to evaluate general geographic variation in service utilisation rather than sex-differentiated spatial processes, sex-specific posterior maps were not created despite sex-specific choropleth maps suggesting variations in crude utilisation patterns. All subsequent interpretations of fixed effects covariates and posterior spatial pattern maps were based on the best-fitting model.

## 3 Ethical clearance

Ethical approval for the CLIMATCH study was obtained from Medical Research Coordinating Committee at the National Institute for Medical Research (NIMR/HQ/R.8a/Vol. IX/4591). Ethical clearance for the PhD study was granted by the Catholic University Health and Allied Sciences and Bugando Medical Centre (CUHAS/BMC) joint Ethics Review Committee (CREC/944/2025).

## 4 Results

### 4.1 Descriptive characteristics of the districts

Table 1 shows clear demographic and healthcare service use differences between rural and urban districts. Urban districts have markedly significantly higher healthcare service utilisation rates for both diabetes and hypertension. Urban healthcare service utilisation rates for hypertension (median 26.8 per 1,000) far exceeded rural healthcare service utilisation rates for hypertension (7.8 per 1,000). Female healthcare service utilisation rates were consistently higher than male healthcare service utilisation rates across both conditions in urban and rural settings. These differences mirror demographic and socioeconomic patterns, with urban districts having a higher literacy rate, greater access to television, and higher mobile phone ownership.

**Table 1:** Descriptive characteristics by rural/urban classification of districts in Tanzania mainland.

Variables	Rural median (IQR)/ Mean ±	Urban median (IQR)/Mean ± SD	*P*-value
Diabetes patients' healthcare utilisation (per 1,000 population)			
National rate	2.4 (1.3–4.1)	13.3 (8.3–21.7)	**<0** **.** **001** ^a^
Female rate	2.5 (1.3–4.6)	15.1 (8.1–23.2)	**<0** **.** **001** ^a^
Male rate	2.3 (1.2–3.7)	11.3 (7.6–20.1)	**<0** **.** **001** ^a^
Hypertension patients' healthcare utilisation			
National rate	7.8 (4.2–16.5)	26.8 (18.9–49.0)	**<0** **.** **001** ^a^
Female rate	11.9 (6.3–24.9)	37.2 (28.3–64.7)	**<0** **.** **001** ^a^
Male rate	6.3 (3.5–14.5)	26.8 (18.3–46.1)	**<0** **.** **001** ^a^
National estimates/rates for explanatory factors			
Literacy rate	79.1 (73.0–86.2)	93.0 (89.2–96.1)	**<0** **.** **001**
Employment rate	78.1 (75.9–80.1)	76.9 (74.5–78.6)	**0** **.** **008** ^a^
Proportion of ownership of Radio	30.0 (25.0–38.9)	41.9 (34.4–47.2)	**<0** **.** **001** ^a^
Proportion of ownership of Television	14.4 (10.1–23.4)	38.1 (28.0–43.7)	**<0** **.** **001** ^a^
Proportion of ownership of mobile phone	79.39 ± 5.15	87.24 ± 4.6	**<0** **.** **001** ^a^
Proportion of health facilities	0.5 (0.4–0.6)	0.4 (0.3–0.7)	**0** **.** **488**
Household size	4.3 (3.8–5.3)	3.8(3.5–4.3)	**<0** **.** **001** ^a^

Bold indicates significant at 5% level of significance.

^a^ Significant at 5% level of significance, IQR, Interquartile Range, SD, Standard Deviation, and the *p*-value represents the statistical comparison between urban and rural districts.

### 4.2 Choropleth maps for health service utilisation

**Figure 1:**
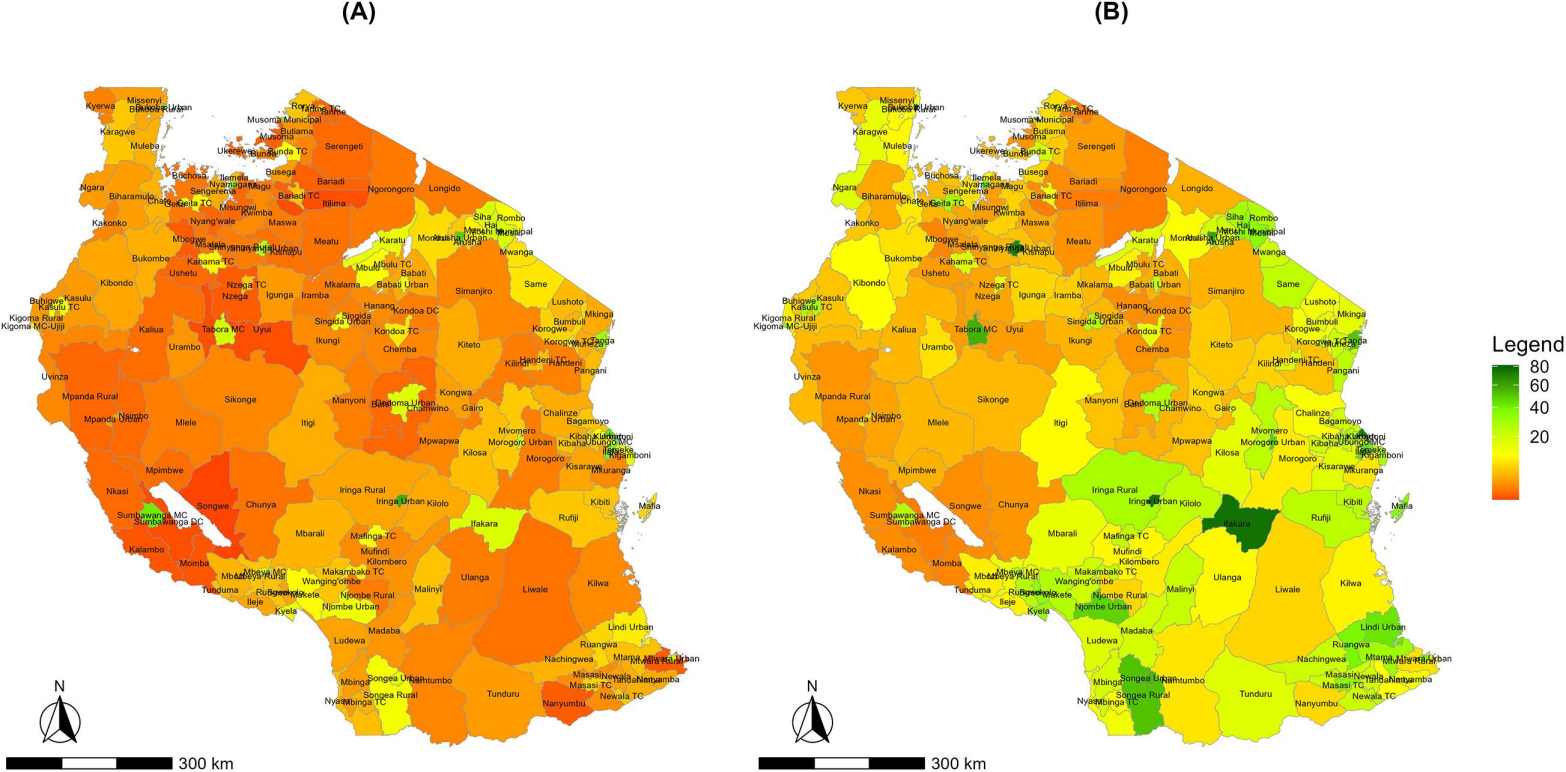
Spatial distribution of district-level healthcare service utilisation rates per 1,000 population for **(A)** diabetes and **(B)** hypertension at the national level in Tanzania mainland, 2024.

Figure 1 shows healthcare service utilisation for both diabetes and hypertension. Spatial variation in healthcare service utilisation for diabetes is evident, with higher rates concentrated in major urban centres such as Arusha urban, Moshi municipal, and Mbeya municipal. Lower rates were observed across rural districts, especially in the central (for example, Bahi and Chemba) and western (such as Urambo) parts of Tanzania. Healthcare service utilisation for hypertension was highest in urbanized and coastal regions, with large geographic disparities across the mainland.

**Figure 2:**
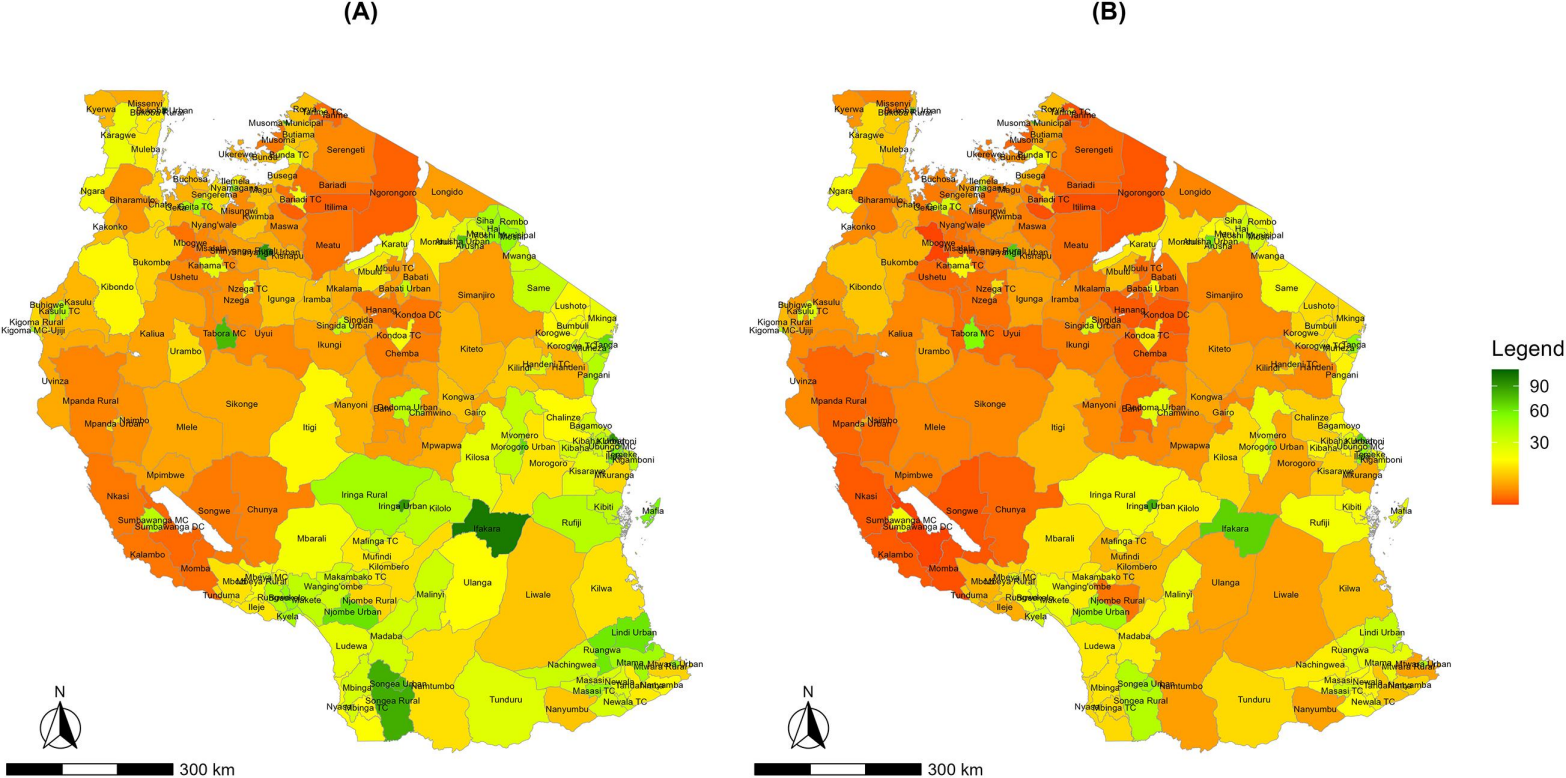
Spatial distribution of district-level hypertension healthcare service utilisation rates per 1,000 population for hypertension by gender in Tanzania mainland, 2024: **(A)** females and **(B)** males.

Figure 2 shows healthcare service utilisation for hypertension stratified by gender. Females had higher and more spatially concentrated healthcare service use, particularly in urban districts such as Dar es Salaam, Ifakara, Arusha urban, and Tabora municipal, compared to males. Lower rates were observed in districts like Uvinza, Chunya, and Ruangwa. Male healthcare service utilisation was comparatively lower and more evenly distributed across regions. Male healthcare service utilisation was highest in a few districts, such as Tanga city council, Kigoma urban, and Moshi municipal. In contrast, districts such as Busega, Bahi, and Kiteto showed substantially lower male healthcare service utilisation. Healthcare utilisation for diabetes was not stratified by gender, as there were no significant differences between the national estimates and the gender stratification.

### 4.3 Spatial clustering

Across the country, local indicators of spatial clustering showed only a limited number of statistically significant hotspots or cold spots between districts, indicating that strong clustering in healthcare service utilisation rates was relatively limited. Global spatial statistics, however, revealed moderate but significant autocorrelation, particularly for hypertension (Table 2). National hypertension healthcare service utilisation exhibited a Moran's I of 0.173 (*p* = 0.003), indicating moderate spatial dependence, while national diabetes healthcare service utilisation showed weaker clustering (I = 0.099, *p* = 0.025). Gender-specific hypertension healthcare service utilisation patterns demonstrated clear spatial differences. Female hypertension service utilisation showed stronger spatial clustering (Moran's I = 0.195, *p*-value = 0.001) than that observed among males (Moran's I = 0.118, *p*-value = 0.01), suggesting that female healthcare utilisation for hypertension was more spatially concentrated. In contrast, diabetes healthcare service utilisation exhibited weaker spatial clustering overall, with statistically significant clustering observed at the national and female levels, but not among males.

**Table 2:** Moran's I and *P*-values for each condition.

Condition	Moran's	*P*-value
Hypertension		
National	0.173	0.003^b^
Gender		
Male	0.118	0.014^a^
Female	0.195	0.001^b^
Diabetes		
National	0.099	0.025^a^
Gender		
Male	0.070	0.067
Female	0.116	0.014^a^

^a^ Significant at 5% level of significance.

^b^ Significant at 1% level of significance.

Local Gi* hot spot analysis further highlighted these differences. For diabetes healthcare service utilisation, hot spots were evident in urban districts such as Kinondoni, Ilala, and Temeke. Several rural districts, particularly in western and central Tanzania, appeared as cold spots, reinforcing pronounced regional differences. For national estimates, hotspots of hypertension healthcare service utilisation were similarly observed in urban and semi-urban districts, including Iringa Urban, Moshi Urban, and the districts of Dar es Salaam. Cold spots, on the other hand, were observed in sparsely populated rural areas. Notably, Kinondoni and Ilala consistently emerged as significant hotspots for hypertension service utilisation, while cold spots were commonly observed in remote districts across western and central regions. Although isolated hotspots were observed, particularly among females, the majority of districts exhibited non-significant Gi* values, underscoring that spatial clustering of service utilisation in Tanzania is localized rather than widespread.

### 4.4 Bayesian modelling

Table 3 presents fixed-effects IRRs for district-level diabetes healthcare service utilisation across four Bayesian models. Combined spatial models reduced DIC from 2940 to 2461, indicating substantial improvement in model fit. The best model was the convolutional model. The diminishing effect sizes after adding spatial components and the flattening of spatial surfaces imply that diabetes healthcare service utilisation differences are strongly explained by measured socioeconomic covariates rather than residual spatial clustering.

**Table 3:** Diabetes Bayesian INLA models.

Covariates	No spatial component	Spatial component (structured)	Random component (unstructured)	Convolutional (structured and unstructured) model
	IRR (95% CrI)	IRR (95% CrI)	IRR (95% CrI)	IRR (95% CrI)
Literacy rate	1.04 [1.03, 1.06]^a^	1.04 [1.03, 1.06]^a^	1.03 [1.02, 1.05]^a^	1.03 [1.01, 1.05]^a^
Employment rate	1.00 [0.97, 1.03]	1.00 [0.97, 1.03]	1.01 [0.98, 1.04]	1.01 [0.97, 1.04]
Proportion of ownership of Radio	0.97 [0.96, 0.98]^a^	0.97 [0.96, 0.98]^a^	0.97 [0.96, 0.98]^a^	0.97 [0.96, 0.99]^a^
Proportion of ownership of Television	1.03 [1.02, 1.05]^a^	1.03 [1.02, 1.05]^a^	1.03 [1.01, 1.04]^a^	1.02 [1.01, 1.04]^a^
Proportion of ownership of mobile	1.06 [1.02, 1.09]^a^	1.05 [1.02, 1.09]^a^	1.08 [1.05, 1.12]^a^	1.10 [1.06, 1.14]^a^
Proportion of health facilities	0.99 [0.74, 1.32]	0.99 [0.74, 1.32]	1.06 [0.77, 1.46]	1.16 [0.83, 1.61]
Household size	0.84 [0.72, 0.97]^a^	0.84 [0.72, 0.97]^a^	0.87 [0.75, 1.01]	0.88 [0.72, 1.07]
Model diagnostics				
DIC	2,940.567	2,940.858	2,507.995	2,461.784
WAIC	2,943.425	2,943.932	2,542.063	2,480.682
p.eff (DIC)	9.039369	10.98156	191.4806	181.9438
p.eff (WAIC)	10.6685	12.0705	192.8969	172.8444

^a^ Significant at 5% level of significance, IRR, Incidence rate ratio; CrI, Credible Interval; DIC, Deviance Information Criterion; WAIC, Watanabe Akaike Information Criterion; p.eff, effective number of parameters.

Literacy rate was a consistently strong positive predictor, suggesting that districts with a higher literacy rate had 3% [incidence rate ratio (IRR) = 1.03; 95% credible interval (CrI): 1.01–1.05] increased rate of healthcare service utilisation for diabetes. Ownership of television was associated with a 2% higher rate of healthcare service utilisation (IRR=1.02; 95%CrI 1.01–1.05) while mobile phones had 10% (IRR = 1.10, 95%CrI: 1.06–1.14) increased rate of diabetes healthcare service utilisation. Radio ownership demonstrates a weak but consistent negative relationship, showing a 3% (IRR = 0.97, 95%CrI: 0.96–0.99) decreased rate of diabetes healthcare service utilisation.

**Figure 3:**
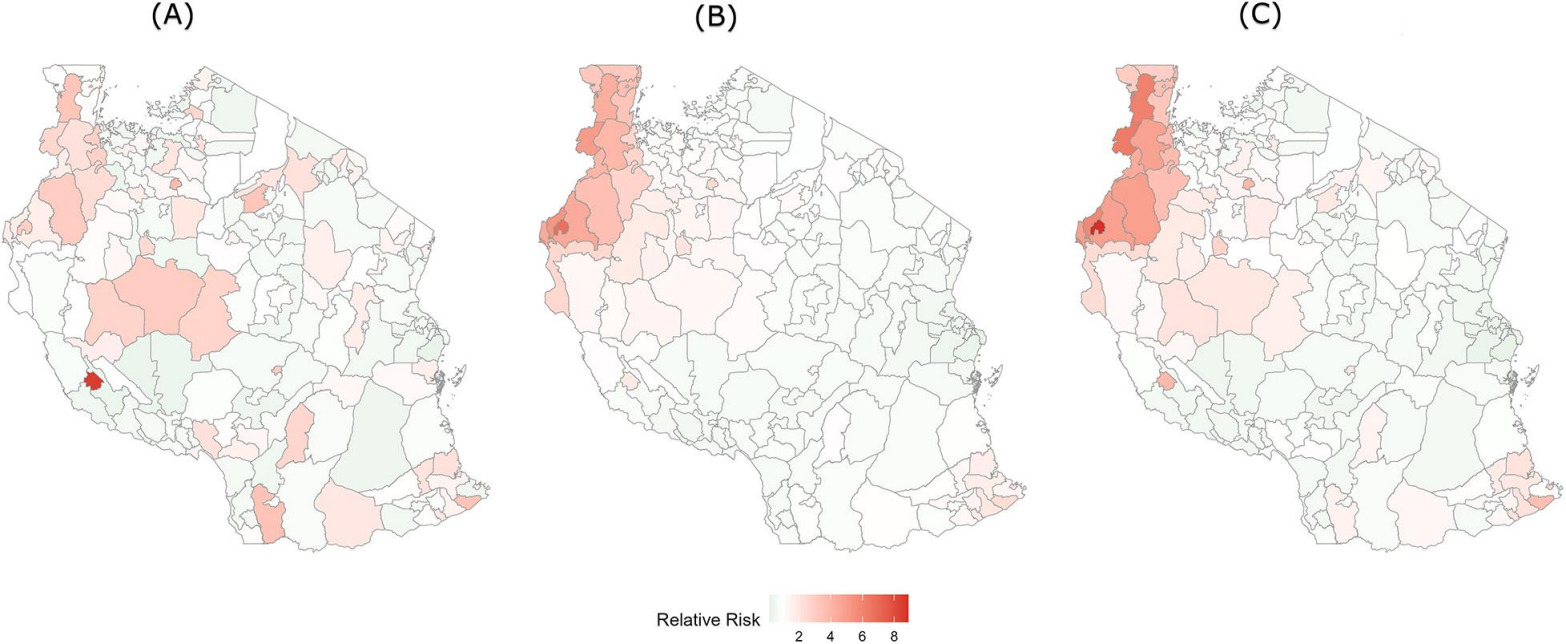
Bayesian spatial effects for diabetes healthcare service utilisation in mainland Tanzania, 2024: **(A)** structured spatial effect representing spatially correlated variation in healthcare service utilisation across neighbouring districts, **(B)** unstructured spatial effect representing district-level random variation not explained by spatial dependence, and **(C)** combined (convolution) spatial effect representing the overall spatial pattern obtained by combining the structured and unstructured components. The legend represents the relative risk of diabetes healthcare service utilisation at the district level compared with the national average, estimated from the Bayesian spatial model. Values greater than 1 indicate higher utilisation relative to the national average, while values below 1 indicate lower utilisation.

In Figure 3, the maps show the structured, unstructured, and combined spatial random effects for diabetes healthcare service utilisation across Tanzania mainland. The structured component showed a largely uniform spatial surface, with only subtle variation between districts. Previously high utilisation urban districts such as Arusha urban, Moshi municipal, and Mbeya municipal did not retain elevated residual spatial effects after adjusting for covariates. The unstructured component displayed mild, random fluctuations with no detectable spatial pattern, and the combined model surface remained nearly flat across all regions except some districts in Kigoma and Kagera. This confirmed the model finding that district-level socioeconomic covariates mainly explained geographic disparities in diabetes healthcare service utilisation, leaving no substantial residual clustering after adjustment.

Table 4 presents IRRs for district-level covariates associated with hypertension healthcare service utilisation. Literacy rate showed a consistent positive association with hypertension service utilisation (IRR = 1.03; 95%CrI: 1.01–1.04), indicating that districts with a higher literacy rate had a 3% increase in healthcare utilisation of hypertension services. Mobile phone ownership emerged as a significant positive predictor (IRR = 1.06; 95%CrI: 1.02–1.10), implying that districts with higher mobile phone ownership had a 6% increase in access to, or engagement with, hypertension services. Household size, on the other hand, remained a strong and consistent negative predictor (IRR = 0.78; 95%CrI: 0.64–0.94), indicating that larger average households had a 22% lower hypertension service utilisation.

**Table 4:** Hypertension Bayesian INLA models.

Covariates	No spatial component	Spatial component (structured)	Random component (unstructured)	Convolutional (structured and unstructured) model
	IRR [95% CrI]	IRR [95% CrI]	IRR [95% CrI]	IRR [95% CrI]
Literacy rate	1.03 [1.02, 1.05]^a^	1.03 [1.02, 1.05]^a^	1.03 [1.01, 1.04]^a^	1.03 [1.01, 1.04]^a^
Employment rate	1.00 [0.98, 1.03]	1.00 [0.98, 1.03]	1.01 [0.98, 1.04]	1.01 [0.98, 1.03]
Proportion of ownership of Radio	0.98 [0.97, 0.99]^a^	0.98 [0.97, 0.99]^a^	0.98 [0.97, 1.00]	0.99 [0.97, 1.00]
Proportion of ownership of Television	1.02 [1.00, 1.03]	1.02 [1.00, 1.03]	1.02 [1.00, 1.03]	1.01 [1.00, 1.03]
Proportion of ownership of mobile phone	1.02 [0.99, 1.06]	1.02 [0.99, 1.06]	1.05 [1.02, 1.09]^a^	1.06 [1.02, 1.10]^a^
Proportion of health facilities	0.95 [0.73, 1.24]	0.95 [0.73, 1.24]	1.10 [0.83, 1.45]	1.13 [0.85, 1.49]
Household size	0.65 [0.57, 0.74]^a^	0.65 [0.57, 0.74]^a^	0.77 [0.64, 0.94]^a^	0.78 [0.64, 0.94]^a^
Model diagnostics				
DIC	3,301.27	3,300.973	3,250.859	2,960.815
WAIC	3,301.556	3,301.279	3,259.491	2,940.609
p.eff (DIC)	9.037079	8.979515	67.06435	192.8386
p.eff (WAIC)	8.757045	8.720383	60.16473	174.3666

^a^ Significant at 5% level of significance, IRR, Incidence rate ratio; CrI, Credible Interval; DIC, Deviance Information Criterion; WAIC, Watanabe Akaike Information Criterion; p.eff, effective number of parameters.

Regarding model performance, the inclusion of spatial random effects improved model fit. The convolutional model had the lowest DIC value (DIC = 2,960), reflecting its superior ability to capture both spatially structured and unstructured residual variation. This supported its selection as the preferred model for inference and mapping of hypertension service utilisation.

**Figure 4:**
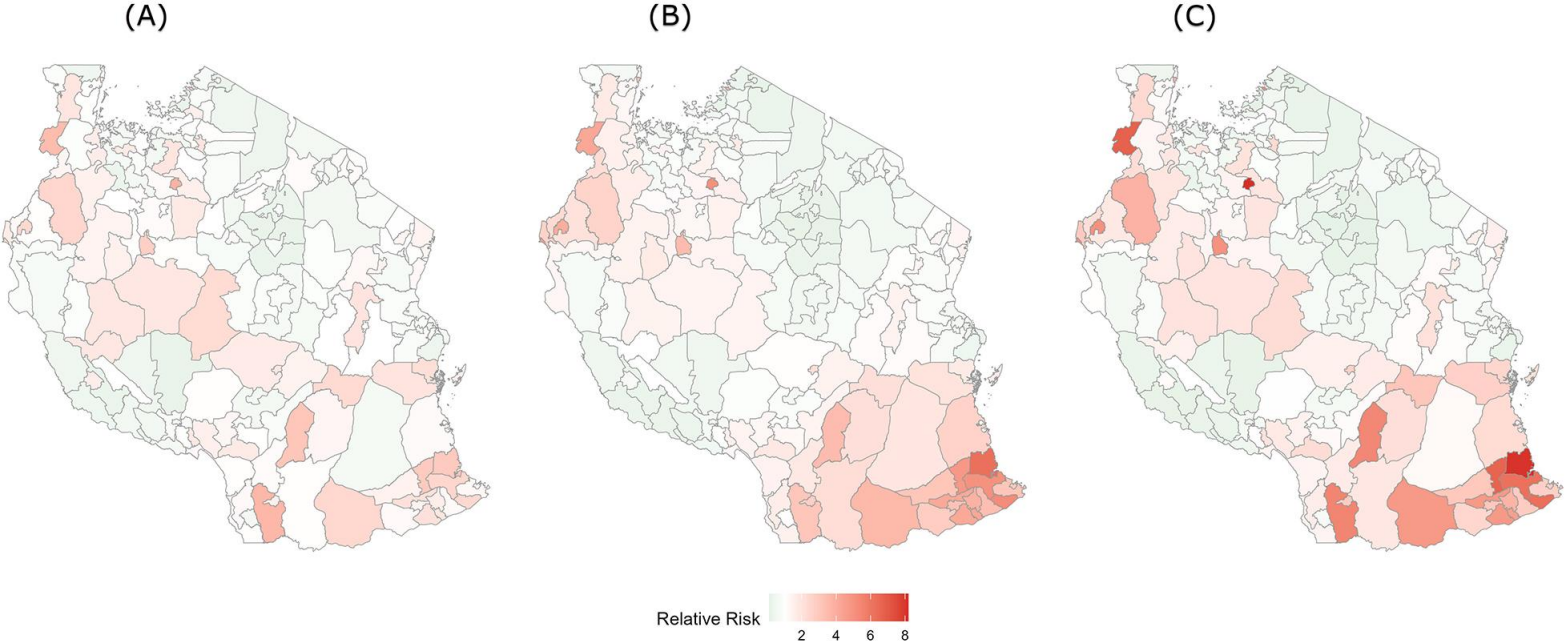
Bayesian spatial effects for hypertension healthcare service utilisation in mainland Tanzania, 2024: **(A)** structured spatial effect representing spatially correlated variation in healthcare service utilisation across neighbouring districts, **(B)** unstructured spatial effect representing district-level random variation not explained by spatial dependence, and **(C)** combined (convolution) spatial effect representing the overall spatial pattern obtained by combining the structured and unstructured components. The legend represents the relative risk of hypertension healthcare service utilisation at the district level compared with the national average, estimated from the Bayesian spatial model.

Figure 4 presents the structured, unstructured, and combined spatial random effects for hypertension healthcare service utilisation across mainland Tanzania. The structured spatial component showed weak spatial dependence, with only a small number of districts exhibiting moderately elevated residual risks, mainly concentrated in parts of the north-western and western zones. High utilisation urban districts such as Kinondoni, Ilala, Arusha urban, and Tabora municipal, that were prominent on crude maps, did not appear as hotspots after adjusting for covariates. Most districts displayed values close to unity, indicating limited remaining spatial autocorrelation after adjustment.

The unstructured component demonstrated scattered, district-specific variability without a clear geographic pattern, suggesting that residual heterogeneity was largely random rather than spatially organised. In the combined spatial effect, a few districts in Kigoma and Kagera, and some southern districts such as Lindi urban, Masasi urban, and Nanyamba retained elevated healthcare service utilisation, while the majority of districts showed near-flat surfaces. Several high-utilisation urban districts evident in crude maps, such as districts in Dar es Salaam municipalities and Arusha urban, did not persist as pronounced residual hotspots after adjustment.

## 5 Discussion

This study mapped and modeled the spatial distribution of healthcare service utilisation for diabetes and hypertension across all districts in mainland Tanzania. The study findings reveal important spatial inequalities in diabetes and hypertension service utilisation across Tanzania. The findings reflect global observations that access to NCD services is strongly shaped by geography, socioeconomic context, and health system preparedness (11, 28, 38–40). Urban districts such as Kinondoni, Moshi urban, and Sumbawanga municipal consistently exhibited high service utilisation, whereas more remote districts, including Tunduru, Sikonge, and Namtumbo, showed low utilisation. These patterns reflect longstanding disparities in healthcare infrastructure, staffing, and diagnostic capacity (12, 41). These differences also reflect disparities in urbanisation and service access.

The spatial disparities observed in healthcare service utilisation for diabetes and hypertension may reflect broader challenges in achieving Universal Health Coverage (UHC) in Tanzania. UHC seeks to ensure that all individuals have access to essential health services, including diagnosis, prevention, treatment, and rehabilitation, without experiencing financial hardship (42). Recent healthcare financing reforms, including the Community Health Fund (CHF) and National Health Insurance Fund (NHIF), aim to expand access to a minimum benefit package that includes outpatient and inpatient services, consultations, diagnostic tests, and essential medicines (43). However, effective coverage for NCDs such as diabetes and hypertension remains uneven in practice. Studies have shown that shortages of laboratory commodities, medicines, and trained personnel continue to constrain the delivery of NCD services in many health facilities, particularly at lower levels of care and in rural areas (27, 44). Furthermore, out-of-pocket payments continue to account for a substantial share of health expenditures, potentially limiting access to health care for some population groups (45). These structural limitations may contribute to the spatial inequalities in healthcare service utilisation observed in this study, as rural districts often face longer travel distances to health facilities, lower diagnostic capacity, and reduced availability of specialised services.

Crude gender-specific maps revealed meaningful spatial differences in service utilisation among hypertensive patients. Higher female healthcare service utilisation aligns with evidence that women often have greater engagement with health systems, often due to gender-sensitive differences in health-seeking behavior and accessibility of long-term care through reproductive health services (46, 47). Lower male service utilisation is consistent with studies showing that cultural norms, occupational patterns, and perceptions of invulnerability reduce health-seeking among men (48). These patterns may reflect gender differences in awareness, access, or health system engagement.

Sociodemographic factors were strongly associated with service utilisation. Literacy and media ownership (radio, television, and mobile phones) consistently increased utilisation, likely due to improved awareness, health information access, and navigation of the health system, findings echoed in earlier work in Sub-Saharan Africa (49, 50). An interesting finding of this study was the negative association between radio ownership and diabetes healthcare service utilisation. This pattern may reflect underlying socioeconomic and geographic differences across districts rather than a direct association between radio ownership and healthcare utilisation. Radio ownership tends to be more common in rural or less economically developed districts, where access to healthcare facilities, diagnostic services, and specialised care for chronic diseases such as diabetes may be limited. In contrast, television and mobile phone ownership are often more prevalent in urban and economically developed districts, where healthcare infrastructure and access to health information are generally stronger. Previous studies have shown that urban populations in low- and middle-income countries tend to have better access to diagnosis and treatment for non-communicable diseases compared with rural populations (11, 51). Therefore, the observed association may reflect broader structural differences in healthcare accessibility and socioeconomic development across districts rather than a direct influence of radio ownership on healthcare-seeking behaviour. The negative influence of household size reflects financial constraints and competing demands in larger households, similar to evidence from Ethiopia and Nigeria (39, 52, 53).

Spatial models improved fit, indicating that geographic context continues to shape NCD service utilisation beyond measured covariates. The findings align with other spatial NCD studies from Uganda, Kenya, and South Africa, which show that distance to healthcare services, regional infrastructure investment, and historical resource allocation patterns drive spatial clustering in service utilisation (46–49).

One of the key findings of this study is that residual spatial autocorrelation in diabetes and hypertension healthcare service utilisation was substantially reduced after adjusting for district-level socioeconomic covariates and health system factors. The structured and combined spatial effects revealed minimal remaining spatial clustering, with only a few districts maintaining high service utilisation. This suggests that measurable district-level factors, rather than unobserved spatial processes, accounted for a significant proportion of the observed geographic disparities in service utilisation. This contrasts with findings from other studies in which unexplained spatial clustering persisted (54, 55). It is important to note that this does not imply an absence of healthcare demand in rural settings, rather, it indicates that variations in healthcare utilisation reflect underlying socioeconomic conditions, access, and health-seeking behavior captured by the covariates included in the model.

The findings of this study have important policy implications. The absence of persistent residual hot spots suggests that interventions should prioritize socioeconomic and access-related factors rather than focusing solely on geographic location. In low-performing districts such as Bahi, Chemba, and Urambo, strengthening healthcare infrastructure, enhancing media-based health education, and addressing socioeconomic disparities could improve healthcare service utilisation.

Broadly, the findings highlight the value of combining robust Bayesian modeling with spatial mapping to extract contextual and structural drivers of healthcare service utilisation. These approaches emphasize the importance of district-level planning and targeted investments in healthcare infrastructure to reduce inequalities in service utilisation. Given its higher prevalence, chronic nature, and greater reliance on routine primary healthcare services, hypertension may be influenced by additional contextual or behavioral factors that are not fully captured by the available district-level covariates. This interpretation is supported by the more pronounced reduction of spatial patterns observed for diabetes compared with hypertension.

## 6 Strengths and limitations

One of the key strengths of this study is the use of DHIS2 data covering all of mainland Tanzania. The use of the DHIS2 dataset enables a comprehensive national analysis rather than regionally limited representations. In addition, DHIS2 reflects real-world service utilisation, making the findings directly applicable to health policy and planning. To the best of our knowledge, this study represents the first spatial mapping of patient service utilisation for diabetes and hypertension in mainland Tanzania to provide fresh insights into the distribution of NCD healthcare utilisation by identifying underserved and high-burden areas, thereby guiding the Ministry of Health and its partners in resource allocation and targeted interventions.

The study employs advanced and robust spatial analytical approaches, offering clear advantages over traditional statistical techniques by explicitly identifying spatial variation in patient healthcare service utilisation for diabetes and hypertension across mainland Tanzania. The findings have important public health implications for improving NCD prevention and control in Tanzania and other low- and middle-income settings. By incorporating spatial random effects, the Bayesian approach reduces bias and mitigates the risk of misleading inferences that may arise when spatial autocorrelation among related factors is ignored.

A key limitation of this study relates to the ecological nature of the analysis, which may lead to ecological fallacy in interpretation. The spatial models were conducted using district-level aggregated data. The outcome and the explanatory variables represent average characteristics of districts rather than individual-level attributes, as a result, the observed associations should be interpreted as relationships at the district level and should not be assumed to reflect associations at the individual level. For instance, while districts with higher literacy rates were associated with higher rates of diabetes healthcare service utilisation, this does not necessarily imply that literate individuals are more likely to utilise healthcare services than individuals with lower literacy levels. Additionally, spatial analyses may be influenced by the Modifiable Areal Unit Problem (MAUP), whereby statistical relationships and spatial clustering patterns may vary depending on the scale of administrative boundaries used for analysis. Therefore, the findings should be interpreted as identifying geographical patterns and contextual determinants of healthcare utilisation, rather than individual behavioural relationships.

As with all cross-sectional studies, this analysis cannot establish causal or temporal relationships between predictors and outcome variables. The analysis was limited to data from 2024; therefore, the results do not capture temporal changes in NCD healthcare service utilisation. Furthermore, inconsistencies between districts, underreporting, and misclassification may affect routine DHIS2 data. Patient service utilisation reflects individuals who sought care rather than the true burden of disease in the community, as many undiagnosed or untreated cases remain outside the healthcare service delivery system. In addition, because updated district-level population estimates were not available at the time of analysis, the use of 2022 census data may introduce minor inaccuracies due to population growth or migration between the two years.

Another limitation relates to the use of routine health facility data obtained from the District Health Information System (DHIS2). While DHIS2 provides valuable administrative data for monitoring healthcare service utilisation, several studies have documented challenges related to data completeness, reporting accuracy, and variability in reporting practices across. These issues may be more pronounced in rural settings where health information systems infrastructure and reporting capacity may be limited. Consequently, the utilisation rates presented in this study should be interpreted with caution, as they may reflect both true differences in healthcare demand and variations in reporting practices.

## 7 Conclusion

This study highlights significant geographical disparities in patient service utilisation for diabetes and hypertension across mainland Tanzania. Rural and peripheral areas were characterized by lower rates of service utilisation, whereas urban areas, including Dar es Salaam, Arusha urban, Tanga city, and Morogoro urban, consistently exhibited higher crude utilisation rates. Variations in service utilisation were strongly associated with contextual factors, including literacy levels, household size, and access to communication technologies such as mobile phones and televisions.

The spatial influence on NCD health-seeking behavior was further demonstrated by the presence of spatial clusters and structured random effects. By adopting a Bayesian hierarchical model, we generated robust estimates that accounted for both observed covariates and unmeasured spatial dependencies. These findings underscore the need for targeted, geographically informed public health initiatives to address disparities in NCD care, particularly among populations living in underserved and rural areas.

## Data Availability

The data analyzed in this study is subject to the following licenses/restrictions: The dataset used in this article is not readily available because it contains sensitive health information and is subject to approval by the Ministry of Health in Tanzania. Requests to access these datasets should be directed to moh.go.tz.
